# A critique of the objective function utilized in calculating the Thrifty Food Plan

**DOI:** 10.1371/journal.pone.0219895

**Published:** 2019-07-22

**Authors:** Angela M. Babb, Daniel C. Knudsen, Scott M. Robeson

**Affiliations:** 1 Ostrom Workshop, Indiana University, Bloomington, IN, United States of America; 2 Department of Geography, Indiana University, Bloomington, IN, United States of America; University of Arkansas, UNITED STATES

## Abstract

The Thrifty Food Plan (TFP) is the basis of benefit allocations within the USDA’s Supplemental Nutrition Assistance Program (SNAP), which administers nearly $70 billion in benefits to over 42 million people annually. To produce the allocation of food within the TFP, the USDA uses a mathematical optimization model that solves for the daily apportionment across various food groups. The model is constrained by nutritional and consumption requirements to produce an “optimal” allocation. Despite the importance of the TFP, the computational solution developed by the USDA has received insufficient attention, with only a handful of articles written on the TFP optimization model. Here, we run three alternative objective functions that are simpler than the one used by USDA. Our first alternative objective function minimizes the sum of squared errors between the consumed market basket of goods and an allocated market basket of goods, the second alternative objective function minimizes the sum of the absolute value of the difference between the consumed market basket of goods and an allocated market basket of goods, and the third alternative objective function minimizes the weighted absolute deviation of allocations and actual consumption expressed as a proportion of observed consumption. A clear theoretical advantage of either of our methods is that they eliminate the need to arbitrarily set allocated consumption to nonzero values, as is the case for the logarithmic objective function used by USDA. In an operational sense, we find that our model formulations produce an allocation that fits actual consumption better than the objective function employed by the USDA.

## Introduction

The Thrifty Food Plan (TFP) is the principal policy lever behind the Supplemental Nutrition Assistance Program (SNAP) [1], a federal food aid program administered under the United States Department of Agriculture (USDA). The USDA calculates four diet plans: the liberal, the moderate-cost, the low-cost, and the thrifty. The TFP is the lowest-cost of the four USDA-designed diet plans and determines the maximum allotment of monetary nutrition assistance to over 42 million Americans [2]. SNAP, which accounts for a majority of the USDA budget, rests on the TFP as an accurate calculation of what it costs the poorest Americans to acquire an adequately nutritious diet.

Despite its incomparable importance for determining federal nutrition assistance, the TFP optimization model has received relatively little academic attention, with only a handful of academic papers written on the topic. Notable exceptions include Wilde and Llobrera’s [1] examination of the TFP in which they argue in favor of models using a least squares approach, and examination of lactose intolerance and constriction of Vitamin E within the TFP framework [3,4]. Still others have focused on the unrealistic amount of time it takes to shop for and prepare menus derived as part of the TFP [5] and the USDA’s Healthy Incentives Pilot (HIP) program [6,7,8]. A recent thorough analysis by Babb [9] of the USDA’s TFP calculation highlights the need to revisit the TFP mathematics to examine the implications of medically necessary diets (lactose intolerance, type 2 diabetes, etc.) and also to rethink the objective function used by the USDA. Because of fortification with vitamins and minerals and a relatively low cost via government subsidies, the official TFP includes more than three servings of liquid milk per person per day–which is atypical of current consumption and not appropriate for individuals with lactose intolerance. Furthermore, Babb [9] argues the form of the objective function works to normalize consumption patterns, obscuring the structural barriers to food access and problematically utilizing consumption as a proxy for palatability.

At its heart, the TFP is a quadratic programming problem containing an objective function that determines an allocation by seeking to minimize the purchase-weighted difference between a given allocation and actual consumption. Constraints are used to ensure adequate caloric and nutritional intake, as well as consumption of the USDA mandated “pyramid” [10]. There is also a budget constraint. For historical and seemingly anachronistic reasons, the objective function applies a logarithmic transformation to both the allocation and to consumption. The logarithmic transformation necessitates having non-zero allocations in all food groups, which creates the problem of requiring consumption for all age groups (e.g., infants must consume non-zero amounts of coffee and tea in the USDA’s TFP solution). Logarithmic transformations also unevenly weight positive and negative deviations from an objective value as positive deviations get reduced more by the transformation than negative deviations, an effect that is amplified when the allocations are close to zero (e.g., |log(1.999)-log(1)| << |log(0.001)-log(1)|). In addition, the logarithmic transformation alters the dimensionality of the variables such that their original units are not preserved in determining the model solution.

The purpose of this commentary is to demonstrate that alternative formulations to the price-weighted logarithmic transformations within the objective function may be preferred. In particular, we hypothesize that objective functions that minimize the weighted difference between a given allocation and actual consumption squared (subject to the same constraints), the weighted absolute value of the difference between a given allocation and actual consumption, and the weighted absolute deviation of allocations and actual consumption expressed as a proportion of observed consumption

- Provide simpler theoretical models for food allocation solutions.
- Are easier to interpret and more parsimonious than the USDA formulation.
- Produce improved goodness-of-fit over the USDA formulation when compared to actual consumption
- Provide less sparse allocations across broad food groups.

## Data and methods

Data for determining our model solutions include actual consumption, cost of purchase, nutrient content, energy content, and contribution to USDA MyPyramid standards for 58 commodities. The data also include upper and lower bounds on nutrient consumption, energy consumption, and contribution to MyPyramid standards, as well as SNAP daily and monthly benefit totals for 17 age/gender groupings of the US population. Data were supplied to us under a Freedom of Information Act request and are from 2001. These data from 2001 were used by the USDA for the TFP calculations of 2006. The TFP was recalculated in summer 2016, but results of this most recent calculation and associated data are not yet available.

### Models

The model used by the USDA may be written as:

$$MIN\;Z=\sum_{i}W_{i}(\ln T_{i}-\ln X_{i}{)}^{2}$$ (1)

where *W*_*i*_ is the relative cost of commodity *i* calculated as $C_{i}T_{i}/\sum_{i}C_{i}T_{i}$, *T*_*i*_ is the actual consumption of commodity *i* and *X*_*i*_ is the TFP allocated amount of commodity *i*. The minimization is subject to the following constraints:

1. $\sum_{i}C_{i}X_{i}\leq B$, where *C*_*i*_ is the cost of a unit of commodity *i* and *X*_*i*_ is the allocated amount of commodity *i*, subject to budget constraint *B* (58 commodities);
2. *L*_*j*_≤*N*_*ij*_*X*_*i*_≤*U*_*j*_ for all commodities *i* and nutrients *j* (58 commodities & 19 nutrients);
3. *L*_*k*_≤*E*_*ik*_*X*_*i*_≤*U*_*k*_ for all commodities *i* and caloric restrictions *k* (58 commodities and 8 caloric restrictions);
4. *L*_*m*_≤*P*_*im*_*X*_*i*_≤*U*_*m*_ for all commodities *i* and pyramid restrictions *m* (58 commodities and 13 pyramid restrictions); and
5. *X*_*i*_≥0 for all commodities *i* (58 commodities).

In constraints 2–4 above we use *L* and *U* to represent lower and upper bounds respectively.

The form of the USDA model can be characterized as a cost-weighted log-log transform model. In this formulation, the log transforms require that every commodity have a nonzero value, which may be unreasonable for certain age groups or diets. Wilde and Llobrera [1] argue that there is no clear rationale for the particular form of objective function used by the USDA and test a sums of squared error formulation (see model (2) below) in their critique of the TFP. Citing Hanson [10], they state (p. 279) the “USDA’s reasonable goal in choosing this function (model (1)) was to assign a greater penalty to food plan choices that fall short of current consumption, while assigning a smaller penalty to food plan choices that exceed current consumption” [1]. Peter Basiotis, supervisor of the 2006 TFP team, provides an alternate reason, stating “the purpose of the logs was to ‘scale’ (or ‘equalize’ the magnitudes of) the variables. That was needed because the algorithm is sensitive to discrepant magnitudes in the variables in terms of convergence. For example, it might be impossible for the algorithm to converge to a solution if the quantities were left in grams, as fluids for example would be much higher than solids” (personal communication 2016). Still, Hanson [10] claims the objective function is “not accomplishing what it was specified to do…”and that “It will be worthwhile reconsidering the specification of the objective function” (p. 19). This lack of clear rationale and, moreover, the lack of confidence in the particular form of the objective function is especially troubling given that considerable preprocessing or post-processing must occur during calculation to account for the occurrence of zero values for some of the commodities [9].

The second model used here is one suggested by Wilde and Llobrera [1] and involves the minimization of sum of squared error terms:

$$MIN\;Z=\sum_{i}W_{i}{\left(T_{i}-X_{i}\right)}^{2}$$ (2)

that is subject to the same five constraints as model (1). Wilde and Llobrera [1] find that such a model may be preferable in that it provides solutions that typically meet the constraints of the TFP while not varying from actual consumption noticeably more that the USDA’s TFP allocation.

We also evaluate a third model that builds on recent work that argues for preserving the actual magnitudes (absolute values) of differences between two vectors of data, as measures that square differences of magnitude effectively weight large deviations more than others [11,12]. This model has the form:

$$MIN\;Z=\sum_{i}W_{i}\left|T_{i}-X_{i}\right|$$ (3)

and also is subject to the same five constraints as model (1). While models (2) and (3) appear visually similar, the minimization in model 3 that uses least absolute values, also known as least absolute deviations (LAD), can produce different solutions from those generated by least squares models (2), as demonstrated by the considerable literature on LAD estimators, e.g. [13, 14, 15].

A final model that we evaluate is widely used in diet optimization studies [16, 17, 18] and minimizes the weighted absolute deviation of allocations and actual consumption expressed as a proportion of observed consumption. An advantage of this approach is that this formulation is insensitive to the units in which food is measured. From a strictly mathematical point of view, a disadvantage is that, should observed consumption of a food category for any age/sex group be zero, the value of model (4) becomes undefined (similarly, food categories with near-zero observed consumption can cause instabilities in the model). The objective of this last model is:

$$MIN\;Z=\sum_{i}W_{i}|(X_{i}-T_{i})/T_{i}|$$ (4)

subject to the five constraints of model (1).

To solve models (2), (3), and (4), we use a nonlinear generalized reduced gradient solution algorithm [19] that is implemented in Microsoft Excel using an add-in created by Frontline Solvers. Multiple start methods were used to ensure the ultimate solution is a global, not local, minimum. Our work focuses on three age groups (14–18, 20–50 and 51–70 year-old males and females), as these capture the preponderance of decision-making consumers on SNAP and thus impacted by the TFP.

Two caveats are in order with respect to our work. First, because we are using specific age/sex ranges, we are able to specify constraint set 3 (governing caloric restrictions) exactly for the age groups, saving computational time. Second, we follow USDA practice in setting the sodium constraint’s upper limit to the higher of US consumption and the upper limit (UL) for sodium established by the 2005 *Dietary Guidelines for Americans* [20]. Only 51–70 year-old females consume less sodium than the UL of 2300 milligrams per day. Using the UL for sodium for age/sex groups other than 51–70 year-old females, no feasible solution to the TFP exists for the USDA model [10] or for the models that we report here.

### Goodness of fit and sparsity measures

We focus on the ability of models (1)–(4) above to recover actual consumption subject to constraints. To assess this aspect of model fit, we use the root mean squared error (RMSE) and mean absolute value error (MAE). While RMSE is widely used and customary for assessing model fit [21], MAE is more apt to linearly evaluate differences between empirically observed and allocated values regardless of the distribution of those errors [22]. Also, without knowing the probability distribution (normality) of the errors, RMSE is less interpretable, especially when comparing different model solutions [22]. Using the notation above:

$$RMSE=\sqrt{\sum_{i}{\left(T_{i}-X_{i}\right)}^{2}/n}$$ (5)

and

$$MAE=\sum_{i}\left|T_{i}-X_{i}\right|/n$$ (6)

where *n* is the number of commodities in the objective function (here, *n* = 58). Neither RMSE nor MAE have exact distributions. In such cases, bootstrap techniques often are used for significance testing. Here, however, this approach makes little sense as the random rearrangement of allocated consumption will, in most cases, produce solution sets that do not meet caloric, nutritional, or dietary balance (so-called “pyramid”) constraints. Furthermore, sampling variability in this modeling framework enters via the observed consumption; however, distributional information within observed consumption by age/sex group is not available.

Sparsity of allocation by models (1)–(4) is also of concern since *ceteris paribus* less sparse allocations are preferred to sparse allocations in terms of providing a well-rounded diet. Calculation of sparsity employs two measures–a simple enumeration of the number of allocations less than one gram and the Gini Coefficient which is calculated using the formula for ordered data:

$$Gini=(2/n^{2}\mu )*\sum_{i}i\left(X_{i}-\mu \right)$$ (7)

where *n* is sample size, *μ* is the mean of the vector *X*, *i* is the rank of *X*_*i*_, and *X*_*i*_ is the *i*th value of a vector sorted in ascending order. Gini is a standard measure of distribution unevenness that has been shown by Hurley and Rickard [23] to be a superior measure for evaluating sparsity as well. The Gini Coefficient has a lower limit of 0 when all values of the vector are equal and a theoretical upper limit of 1 when all values except one are zero (in an infinitely large population). Similar to arguments above, significance testing or confidence intervals for Gini Coefficients are not used here because they require resampling methods that produce inappropriate solution sets.

## Results

An examination of Table 1 indicates that, based on the goodness-of-fit measures applied, the models (2–4) all appear to be superior to the USDA model for all age/sex groups studied. Furthermore, based on goodness-of-fit alone, models (3) and (4) are typically superior to (2). For 51–70 year olds, models (2) and (3) are superior to (4).

**Table 1:** Goodness of fit measures for models (all units are 100 grams).

**14–18 Year-Old Males**	**RMSE**	**MAE**
USDA (Model 1)	1.68	0.76
Minimum Squared Error (Model 2)	1.20	0.67
Minimum Absolute Value Error (Model 3)	1.34	0.55
Minimum Absolute Proportions (Model 4)	1.35	0.55
**14–18 Year-Old Females**		
USDA (Model 1)	1.34	0.56
Minimum Squared Error (Model 2)	1.18	0.54
Minimum Absolute Value Error (Model 3)	1.12	0.49
Minimum Absolute Proportions (Model 4)	1.12	0.49
**20–50 Year-Old Males**		
USDA (Model 1)	1.50	0.72
Minimum Squared Error (Model 2)	1.29	0.64
Minimum Absolute Value Error (Model 3)	1.18	0.59
Minimum Absolute Proportions (Model 4)	1.22	0.58
**20–50 Year-Old Females**		
USDA (Model 1)	1.44	0.66
Minimum Squared Error (Model 2)	1.29	0.61
Minimum Absolute Value Error (Model 3)	1.29	0.58
Minimum Absolute Proportions (Model 4)	1.29	0.58
**51–70 Year-Old Males**		
USDA (Model 1)	1.42	0.66
Minimum Squared Error (Model 2)	0.81	0.44
Minimum Absolute Value Error (Model 3)	0.95	0.48
Minimum Absolute Proportions (Model 4)	1.12	0.53
**51–70 Year-Old Females**		
USDA (Model 1)	1.39	0.67
Minimum Squared Error (Model 2)	0.85	0.47
Minimum Absolute Value Error (Model 3)	0.90	0.45
Minimum Absolute Proportions (Model 4)	1.18	0.56

An additional property of the solutions is that, in all cases except 51–70 year old females, model (2) delivers solutions that are sparser than models (3) and (4) when using the number of food group allocations less than one gram as the measure of sparsity (Table 2). Except for 14–18 year old females and 51–70 old males, all alternative models are less sparse than the USDA model (1). Use of the Gini Coefficient yields a slightly different result. In this case, model (2) universally yields a more uneven (high Gini Coefficient) distribution of allocations across the 58 food groups, while model (4) generally (but not universally) provides the most even distribution (i.e., low Gini Coefficient).

**Table 2:** Sparsity of allocations: Food groups having allocations less than 1 gram & Gini Coefficient.

**14–18 Year-Old Males**	**Allocations < 1 Gram**	**Gini Coefficient**
Actual Consumption	5	0.34
USDA (Model 1)	36	0.38
Minimum Squared Error (Model 2)	42	0.53
Minimum Absolute Value Error (Model 3)	36	0.25
Minimum Absolute Proportions (Model 4)	23	0.25
**14–18 Year-Old Females**		
Actual Consumption	6	0.13
USDA (Model 1)	24	0.25
Minimum Squared Error (Model 2)	41	0.29
Minimum Absolute Value Error (Model 3)	36	0.25
Minimum Absolute Proportions (Model 4)	37	0.25
**20–50 Year-Old Males**		
Actual Consumption	0	0.26
USDA (Model 1)	31	0.37
Minimum Squared Error (Model 2)	39	0.59
Minimum Absolute Value Error (Model 3)	35	0.40
Minimum Absolute Proportions (Model 4)	17	0.27
**20–50 Year-Old Females**		
Actual Consumption	4	0.23
USDA (Model 1)	33	0.26
Minimum Squared Error (Model 2)	42	0.32
Minimum Absolute Value Error (Model 3)	25	0.23
Minimum Absolute Proportions (Model 4)	33	0.23
**51–70 Year-Old Males**		
Actual Consumption	2	0.29
USDA (Model 1)	9	0.32
Minimum Squared Error (Model 2)	34	0.55
Minimum Absolute Value Error (Model 3)	30	0.34
Minimum Absolute Proportions (Model 4)	21	0.22
**51–70 Year-Old Females**		
Actual Consumption	2	0.25
USDA (Model 1)	30	0.32
Minimum Squared Error (Model 2)	35	0.48
Minimum Absolute Value Error (Model 3)	36	0.42
Minimum Absolute Proportions (Model 4)	27	0.25

While our principal goal is to assess the extent to which alternative objective functions provide easier to interpret and more parsimonious solutions than the USDA formulation, we also examine how the alternative models would provide dietary alternatives. Table 3, which provides actual consumption and allocations for 20–50 year old females, illustrates how the allocation models differ across food groups in a situation in which sparsity (as measured by Gini Coefficient) differs relatively little (i.e., for 20–50 year-old females). All allocations are healthier than actual consumption, and include lower amounts of soft drinks and zero amounts of added sugars. In addition, all model allocations call for more legumes, nuts, whole grains, vegetables, and fruits in the diet.

**Table 3:** Actual consumption and allocations (in hectograms) for 20–50 year old females across broad food groupings.

Food Group	Actual	Model 1	Model 2	Model 3	Model 4
Milk	0.63	0.00	0.00	1.08	1.08
Low fat milk	0.71	7.15	6.84	5.61	5.61
Cheese	0.14	0.00	0.00	0.00	0.00
Milk-based desserts	0.30	0.00	0.00	0.00	0.00
Low fat milk-based desserts	0.09	0.00	0.00	0.36	0.36
Low cost red meat	0.09	0.00	0.00	0.00	0.00
Regular cost red meat	0.10	0.00	0.00	0.00	0.00
Low cost lean red meat	0.01	0.30	0.00	0.00	0.00
Regular cost lean red meat	0.11	0.00	0.00	0.00	0.00
Low cost fish	0.03	0.11	0.00	0.00	0.00
Regular cost fish	0.07	0.00	0.00	0.00	0.00
Low cost lean fish	0.00	0.18	0.00	0.00	0.00
Regular cost lean fish	0.06	0.00	0.00	0.00	0.00
Low cost poultry	0.05	0.00	0.00	0.00	0.00
Regular cost poultry	0.14	0.00	0.00	0.00	0.00
Low cost lean poultry	0.02	0.84	0.00	0.00	0.00
Regular cost lean poultry	0.15	0.00	0.00	0.00	0.00
Lunch meat	0.10	0.00	0.00	0.00	0.00
Low fat lunch meat	0.14	0.00	0.00	0.00	0.00
Eggs	0.26	0.00	0.00	0.00	0.00
Meat mixtures	0.48	0.00	0.00	0.00	0.00
Low fat meat mixtures	0.50	0.00	0.00	0.00	0.00
Legumes	0.27	1.05	1.00	1.00	1.00
Nuts and seeds	0.05	0.30	0.93	0.94	0.94
Whole grain breads	0.01	0.30	1.36	1.20	1.20
Non-whole grain breads	0.75	0.00	0.00	0.32	0.32
Non-whole grain cereals	0.10	0.00	0.00	0.00	0.00
Whole grain low calorie cereals	0.02	0.00	0.54	0.26	0.26
Whole grain cereals	0.14	0.56	0.00	0.13	0.12
Whole grain rice and pasta	0.06	2.18	0.00	0.09	0.09
Non-whole grain rice and pasta	0.34	0.78	1.23	0.82	0.82
Whole grain cakes and pies	0.01	0.00	0.00	0.00	0.00
Non-whole grain cakes and pies	0.38	0.00	0.00	0.00	0.00
Whole grain snacks	0.05	0.00	0.00	0.00	0.00
Non-whole grain snacks	0.11	0.42	0.00	0.00	0.00
Grain mixtures	0.98	0.00	0.00	0.00	0.00
Low fat grain mixtures	0.52	0.00	0.00	0.00	0.00
Citrus, melon and berry juice	0.54	0.01	0.00	0.79	0.79
Citrus, melon and berries	0.15	0.00	1.78	1.02	1.02
Other fruit juice	0.41	0.29	0.37	0.79	0.79
Other fruits	0.47	2.70	1.02	0.99	0.99
Potatoes	0.36	0.00	0.00	0.00	0.00
Low fat potatoes	0.16	1.22	1.44	1.32	1.32
Dark green vegetables	0.04	0.24	0.00	0.00	0.00
Orange vegetables	0.01	0.00	0.00	0.00	0.00
Dark green vegetables, no fat	0.03	0.36	0.55	0.55	0.55
Orange vegetables, no fat	0.06	0.62	1.72	1.76	1.76
Other vegetables	0.16	0.00	0.00	0.00	0.00
Tomatoes	0.02	0.90	1.53	1.10	1.10
Other vegetables, no fat	0.27	0.02	0.00	0.00	0.00
Tomatoes, no fat	0.13	0.01	0.00	0.36	0.36
Mixed vegetables	0.04	0.59	0.00	0.10	0.10
Mixed vegetables, no fat	0.09	0.20	0.00	0.24	0.24
Fats and oils	0.27	0.60	0.38	0.29	0.29
Coffee	4.17	0.44	2.08	0.50	0.50
Soft drinks	6.69	0.01	0.80	0.00	0.00
Low calorie soft drinks	1.20	0.00	0.00	0.32	0.32
Sugars	0.25	0.00	0.00	0.00	0.00

Actual consumption is from NHANES 2001–2002; Model 1 is the USDA calculation; Model 2 is minimum squared error; Model 3 is minimum absolute value; Model 4 is minimum absolute proportions.

Results for Females 51–70 varied from the other age/sex groups in terms of goodness-of-fit and sparsity, yet the allocations of food groupings across models show similar patterns (see Table 4). All allocations have less soft drinks than actual consumption and no added sugars. Low fat milk is prominent in all models and more than four times actual consumption levels. In addition, allocations include more legumes, nuts and seeds, whole grain rice and pasta, and more citrus melons and berries than actual consumption.

**Table 4:** Actual consumption and allocations (in hectograms) for 51–70 year old females across broad food groupings.

Food Group	Actual	Model 1	Model 2	Model 3	Model 4
Milk	0.59	0.01	1.48	1.52	0.70
Low fat milk	1.14	7.17	5.44	5.27	6.18
Cheese	0.15	0.00	0.00	0.00	0.00
Milk-based desserts	0.52	0.00	0.00	0.00	0.00
Low fat milk-based desserts	0.27	0.01	0.00	0.24	0.00
Low cost red meat	0.16	0.07	0.00	0.00	0.00
Regular cost red meat	0.11	0.00	0.00	0.00	0.00
Low cost lean red meat	0.05	0.22	0.00	0.04	0.00
Regular cost lean red meat	0.20	0.01	0.00	0.00	0.00
Low cost fish	0.00	0.00	0.00	0.00	0.00
Regular cost fish	0.17	0.01	0.00	0.01	0.00
Low cost lean fish	0.01	0.29	0.00	0.01	0.00
Regular cost lean fish	0.02	0.00	0.00	0.02	0.00
Low cost poultry	0.05	0.00	0.00	0.00	0.00
Regular cost poultry	0.09	0.00	0.00	0.00	0.00
Low cost lean poultry	0.01	0.78	0.00	0.00	0.00
Regular cost lean poultry	0.19	0.01	0.00	0.00	0.00
Lunch meat	0.08	0.00	0.00	0.00	0.00
Low fat lunch meat	0.09	0.00	0.00	0.01	0.00
Eggs	0.26	0.01	0.00	0.00	0.00
Meat mixtures	0.38	0.01	0.00	0.00	0.00
Low fat meat mixtures	0.47	0.01	0.00	0.00	0.00
Legumes	0.32	1.20	1.00	1.00	1.00
Nuts and seeds	0.09	0.29	0.88	0.84	0.93
Whole grain breads	0.02	0.28	0.80	0.90	0.90
Non-whole grain breads	0.76	0.02	0.21	0.28	0.51
Non-whole grain cereals	0.17	0.00	0.00	0.00	0.00
Whole grain low calorie cereals	0.15	0.14	0.12	0.20	0.00
Whole grain cereals	0.16	0.01	0.00	0.03	0.00
Whole grain rice and pasta	0.02	2.67	1.77	0.97	1.14
Non-whole grain rice and pasta	0.65	1.36	1.00	1.16	0.67
Whole grain cakes and pies	0.03	0.00	0.00	0.00	0.00
Non-whole grain cakes and pies	0.27	0.00	0.00	0.00	0.00
Whole grain snacks	0.06	0.00	0.00	0.00	0.00
Non-whole grain snacks	0.11	0.28	0.00	0.00	0.00
Grain mixtures	0.51	0.01	0.00	0.00	0.00
Low fat grain mixtures	0.32	0.00	0.00	0.00	0.00
Citrus, melon and berry juice	0.64	0.03	0.79	0.82	0.86
Citrus, melon and berries	0.46	2.70	1.48	1.16	0.84
Other fruit juice	0.17	0.27	0.37	0.95	1.36
Other fruits	0.61	0.45	0.83	0.76	0.76
Potatoes	0.23	0.91	0.00	0.16	0.00
Low fat potatoes	0.34	0.01	0.78	1.12	1.18
Dark green vegetables	0.11	0.01	0.00	0.11	0.42
Orange vegetables	0.03	0.00	0.00	0.03	0.00
Dark green vegetables, no fat	0.16	1.19	1.63	1.54	1.50
Orange vegetables, no fat	0.08	0.20	0.18	0.75	0.28
Other vegetables	0.28	0.00	0.00	0.00	0.00
Tomatoes	0.23	1.08	1.29	0.96	0.92
Other vegetables, no fat	0.67	0.01	0.11	0.00	0.67
Tomatoes, no fat	0.33	0.01	0.17	0.71	0.14
Mixed vegetables	0.14	1.45	0.87	0.54	0.84
Mixed vegetables, no fat	0.05	0.00	1.16	0.23	0.00
Fats and oils	0.32	0.01	0.00	0.05	0.00
Coffee	7.26	0.76	6.87	0.81	5.57
Soft drinks	2.90	0.02	0.00	0.00	0.00
Low calorie soft drinks	1.89	0.02	1.23	0.49	1.81
Sugars	0.21	0.00	0.00	0.00	0.00

Actual consumption is from NHANES 2001–2002; Model 1 is the USDA calculation; Model 2 is minimum squared error; Model 3 is minimum absolute value; Model 4 is minimum absolute proportions.

## Conclusions

The Thrifty Food Plan (TFP) is the principal policy lever behind the Supplemental Nutrition Assistance Program (SNAP), a federal food aid program administered under the United States Department of Agriculture (USDA) that affects 42 million Americans. Despite its importance, it has received surprisingly little attention from academics. The TFP is a quadratic programming problem containing an objective function that determines an allocation by seeking to minimize the purchase-weighted difference between the logarithmic transformation of a given allocation and actual consumption subject to constraints that ensure adequate caloric and nutritional intake, a federally-mandated budget, and consumption of the USDA mandated “pyramid” [10]. There is also a budget constraint.

The purpose of this research is to demonstrate that several alternative formulations to the USDA’s TFP model are theoretically and computationally more straightforward, are easier to interpret, have improved goodness-of-fit to current consumption, and feature allocations that are not substantially sparser than the current model (1). In particular, models (2), (3), and (4) provide better fit to current consumption than the USDA model (1) for all sex/age groups examined. Models (3) and (4) are often no more sparse than model (1), with Gini Coefficients that usually are less than or equal to that of model (1).

If the main objective is to find a nutritious diet that is *closest* to actual/current consumption patterns, model (3) is most commonly superior across all age/sex groups with the best goodness-of-fit overall. Yet there are a few exceptions, namely coffee, which has relatively low allocations in model (3) compared to actual consumption and the other model solutions. However, if the main objective is to find a well-rounded (i.e. least sparse) diet that is also closest to current consumption patterns, all models have less sparsity than the USDA model, and model (4) generally has the least sparsity.

In closing, we caution that while our modeling results show the value of alternative approaches to calculating the TFP (and thus for examining the cost of existing diets), these results should not be mistaken for proposals of dietary change in a population. There is no objective criteria to determine what would be acceptable as an optimal dietary change for a population.

## Supporting information

S1 Data Data used in allocation models. (XLSX)
